# The Antioxidant Phytochemical Schisandrin A Promotes Neural Cell Proliferation and Differentiation after Ischemic Brain Injury

**DOI:** 10.3390/molecules26247466

**Published:** 2021-12-09

**Authors:** Wentian Zong, Mostafa Gouda, Enli Cai, Ruofeng Wang, Weijie Xu, Yuming Wu, Paulo E. S. Munekata, José M. Lorenzo

**Affiliations:** 1 Kunming Health Vocational College, Kunming 650607, China; zwt366100@zju.edu.cn (W.Z.); xuwj@mail.ynu.edu.cn (W.X.); 2 College of Biosystems Engineering and Food Science, Zhejiang University, Hangzhou 310058, China; 3 Department of Nutrition & Food Science, National Research Centre, Giza 12622, Egypt; 4 College of Nursing, Yunnan University of Chinese Medicine, Kunming 650500, China; enlicaitcm@sina.com; 5 College of health, Yunnan Technology and Business University, Kunming 651701, China; fionanawang@sina.com; 6 Centro Tecnológico de la Carne de Galicia, Rúa Galicia No. 4, Parque Tecnológico de Galicia, San Cibrao das Viñas, 32900 Ourense, Spain; jmlorenzo@ceteca.net; 7 Área de Tecnología de los Alimentos, Facultad de Ciencias de Ourense, Universidad de Vigo, 32004 Ourense, Spain

**Keywords:** neural progenitor cells (NPCs), schisandrin A, ischemic brain injury, stroke, neural regeneration, cell division control protein 42 (Cdc42)

## Abstract

Schisandrin A (SCH) is a natural bioactive phytonutrient that belongs to the lignan derivatives found in *Schisandra chinensis* fruit. This study aims to investigate the impact of SCH on promoting neural progenitor cell (NPC) regeneration for avoiding stroke ischemic injury. The promoting effect of SCH on NPCs was evaluated by photothrombotic model, immunofluorescence, cell line culture of NPCs, and Western blot assay. The results showed that neuron-specific class III beta-tubulin (Tuj1) was positive with Map2 positive nerve fibers in the ischemic area after using SCH. In addition, Nestin and SOX2 positive NPCs were significantly (*p* < 0.05) increased in the penumbra and core. Further analysis identified that SCH can regulate the expression level of cell division control protein 42 (Cdc42). In conclusion, our findings suggest that SCH enhanced NPCs proliferation and differentiation possible by Cdc42 to regulated cytoskeletal rearrangement and polarization of cells, which provides new hope for the late recovery of stroke.

## 1. Introduction

Stroke is a very serious disabling disease, and there is no effective therapeutic strategy at present for this disorder [1]. As an alternative, promoting neurogenesis has been considered as a potential preventive action against neurodegenerative lesions after stroke [2]. However, there are still questions coming along with neuronal regeneration after such ischemia severe of stroke [3]. In this context, NPCs are one of the best ways to enhance the recovery of these kinds of diseases. NPCs are self-renewing and multipotent cells that generate three major cell types in the brain called oligodendrocytes, neurons, and astrocytes [4]. During brain injury, NPCs proliferate and generate new neurons to compensate for cell damage. At present, transplantation of NPCs to treat stroke still needs a lot of in-depth research further and the most feasible method to enhance neuronal repair is promoting endogenous NPCs proliferation migration [5,6].

**Figure 1:**
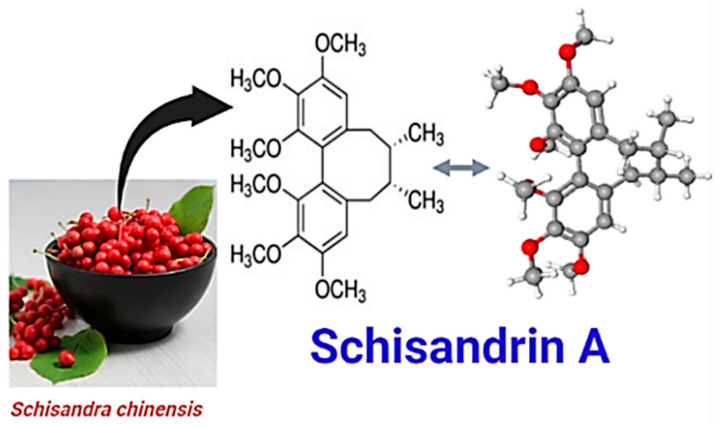
*Schisandra chinensis* fruit and SCH chemical structure.

A variety of pathways are necessary for NPC proliferation and differentiation. These pathways including fibroblast growth factors (FGFs) and epidermal growth factor (EGF) that lead to the activation of intrinsic factors, such as extracellular signal-regulated kinase (ERK) and phosphatidylinositol-3 kinase (PI3K) [7]. Some therapeutic chemicals have the potential to modulate these pathways to promote self-renewal and proliferation of the brain cells. Among these, naturally extracted phytochemicals have been reported to promote NPC proliferation and other related cell tissues in the human body [4,8]. The potential impacts of natural phytochemicals for promoting health and even the food industry are well known [9,10,11,12]. For example, Cai, et al. [13] reported the beneficial impact of herbal medicine containing *Schisandra chinensis* for the treatment of memory impairment or deficits in mice. They also mentioned that the biological and pharmacological effects of *S. chinensis* were due to its dibenzocyclooctadiene-type lignans. Schisandrin A (SCH; Figure 1) is a bioactive lignin compound isolated from *S. chinensis* fruit (Figure 1) and it is widely used in traditional medicine, specifically neurological disorders like senile dementia. Also, it could pass the blood-brain barrier, belonging to biphenyl cyclooctene lignans [14].

Szopa, et al. [15] mentioned that SCH has a favorable function in preventing memory impairment. In addition, SCH has been repeatedly proven to be able to alleviate brain damage caused by oxidative stress [16]. A recent mechanistic study revealed that SCH isolated from activated critical kinase cascades related to endogenous neurogenesis [16]. Moreover, Lee et al. [4] reported that a mechanistic study showed the potential correlation between bioactive antioxidant phytochemcials and NPC regeneration is a result of their critical impact on kinase cascade activation that significantly activates endogenous neurogenesis. Besides, SCH exerts neuroprotective activity by regulating neuroinflammation and ameliorating memory deficits in Alzheimer’s and Parkinson’s disease [17]. Also, the other bioactive potentials of SCH like its in vivo antimicrobial activity against pathogenic gut bacteria [18] and its protective impact on intestinal epithelial cells [19] have made its application in functional food additives of interest for its high activity in the therapeutic intervention of neural disorders. Thus, this novel health-related natural molecule could be used in different medicinal food applications [20,21].

In the present study, we evaluated the capacity of SCH to promote NPC proliferation and differentiation (both in vivo by mice experiments and in vitro by cell line culture of NPCs), through activation of brain cells and neurological functional recovery after stroke. Our results suggest that SCH may serve as a therapeutic agent to promote neurogenesis after ischemic brain injury and other brain disorders. The potential use of SCH as a phytochemical food supplement in medicinal foods for patients who are suffering from stroke and other related diseases will open the field towards novel natural and functional food formulas for specific target diseases.

## 2. Results and Discussion

### 2.1. Neural Progenitor Cell (NPC) Regeneration after Cerebral Ischemia Caused by Photothrombosis

**Figure 2:**
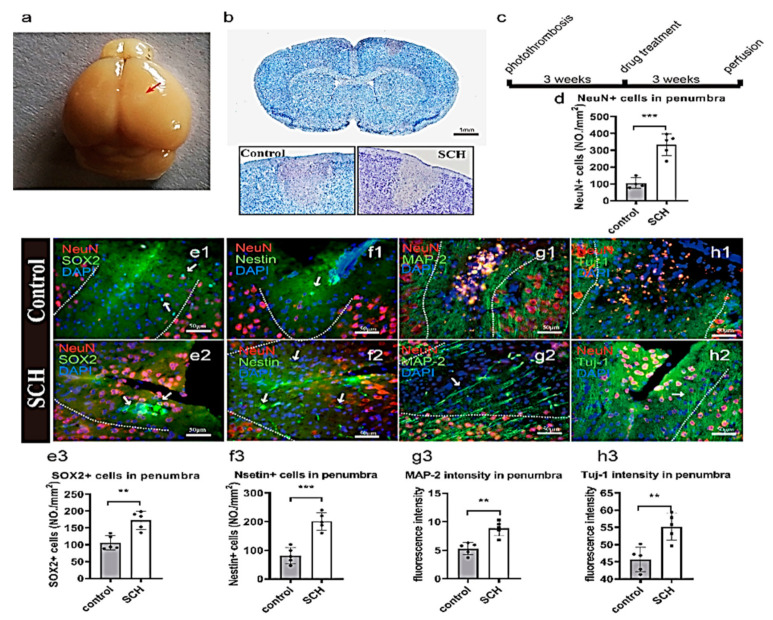
SCH promoting neural regeneration in stroke model by photothrombosis. (**a**) Stroke model by photothrombosis. (**b**) Nissl’s staining of brain ischemic area (grey) at 48 h. (**c**) Schedule of experimental manipulations, scale bar: 1 mm. (**d**) Quantification of the number of NeuN+ neurons in the ischemic area. SOX2 (green), nestin (green), NeuN (red), DAPI (blue), scale bar: 50 μm; (**e1**) The hscattered SOX2 positive cells in the ischemic and core areas of the control group, (**e2**) SOX2 positive NPCs in SCH; (**f1**,**f2**) The number of nestin-positive cells; (**e3**,**f3**) The number of Nestin and SOX2 positive progenitor cells in SCH group was higher than that in the control group. (**g1**,**g2**,**h1**,**h2**) Nerve fiber regeneration in the ischemic area. MAP-2 (green), Tuj-1 (green), NeuN (red), DAPI (blue); (**g3**,**h3**) MAP-2 and Tuj-1 nerve fibers axon or dendrites grow into penumbra in SCH group. The nerve fibers density in SCH group was higher than that in the control group. Data are expressed as the mean ± SEM, and were analyzed by *t* test N = 5, ** *p* < 0.01, *** *p* < 0.001.

In the present study, a photosensitive molecule called Rose Bengal and laser irradiation through the skull was used to cause a model ischemic injury in the cortex [6]. Figure 2a presents the ischemia area (bright area) after the photothrombotic model. The discolored area obtained from Nissl’s staining shows the ischemia (neural loss) area (Figure 2b). Also, immunofluorescence staining of the neuronal nuclei (NeuN) showed the loss of neurons in the ischemic area (Figure 2e–h, Supplementary Material Figure S1a,d). The above validation confirmed the success of the ischemic model implementation. Supplementary Material Figure S1 clearly shows the penumbra (GFAP+) and core (Iba1+) areas at 48 h. Buscemi, et al. [22] mentioned that the major changes in mouse brain microglial morphology in the peri-lesion takes some time that starts after at least 48 h post-injury.

To investigate long-term neural regeneration, SCH was intraperitoneally injected for 3 weeks after inducing cerebral ischemia, and then perfused brain tissue was collected to study the effect of this natural compound on the number of regenerated NPCs. There were scattered SOX2 positive cells in the ischemic and core areas of the control group (Figure 2e1). In SCH group, SOX2 positive NPCs were found, surrounded by differentiated neurons (Figure 2e2). The number of Sox2 positive cells in the SCH group was significantly higher than in the control group (*p <* 0.001; Figure 2e3). Lindvall and Kokaia [23] mentioned that SOX2 is a well-known self-renewal and differentiation stem cell marker. It is widely accepted that the expression of SOX2 is correlated with progenitor cells to functionally mature neuronal cells and glial cells [24,25,26].

Moreover, in the control, there were almost no nestin-positive cells (Figure 2f1). Differently, the SCH group showed a large number of nestin-positive cells (*p* < 0.001; Figure 2f2). Jimenez-Gonzalez, et al. [27] mentioned that nestin is a specific marker for neural stem cells’ (NSCs) self-renewal and ability to differentiate into neuronal cells or glial cells. From these results, we can conclude that SCH promotes the growth of NPCs in vivo. Also, the number of NPCs were increased, which promotes neural differentiation and maturation as well as nerve fiber regeneration. Microtubule-associated protein 2 (MAP-2, a mature neuron marker), is well known to be abundantly expressed in axons and dendrites of neuronal cells [28]. Moreover, in this study, DCX was the marker of the newborn neurons, the NEUN and DCX show the newborn neuron. And we used the tuj-1 and MAP2 for showing nerve regeneration [6].

In this study, we found that from the NeuN positive cells that there were more neurons in the ischemic area of mice injected SCH (Figure 2e–h). This result suggests that SCH may have promoted neurogenesis in the ischemic area. The migration of NPCs to the ischemic area is been considered a promising cell therapeutic strategy against stroke [29]. It is generally found that the neural regeneration capacity of the cortex was weak, in which a few studies have pointed out the existence of NPCs in the cerebral cortex.

In comparison with the control group, SCH group had more MAP-2 positive nerve axon regeneration in the penumbra and core (Figure 2g1,g2). Tuj-1 positive new nerve fibers also invade the penumbra and core (Figure 2h2). In the control group stroke core area, there was neither Tuj-1 positive expression nor MAP-2 expression (Figure 2g2,h2). Conversely, the intensity of MAP-2 and Tuj-1 was significantly higher in the SCH group than that of the control group in the ischemic and core areas (*p* = 0.0016, Figure 1g3; *p* = 0.0038, Figure 1h3). Besides, the NeuN+ neurons in the ischemic and core areas quantified the significant impact of SCH on the stroke area. In which, SCH showed a highly significant difference (*p <* 0.001) compared to the control (Figure 2e2,d). Also, a large number of NeuN positive neurons in the SCH group in the center of the ischemic and core area was observed (Figure 2e3–h3). The best explanation of this phenomena was provided by Zhang, et al. [30] who reported that the significant impact of the phytochemicals on increasing the number of NeuN-positive cells is coming from their influences on the cell’s mitochondria signaling pathways that regulating the activation of pro-caspase-3 into active caspase-3 (cleaved caspase-3). Meanwhile, SCH increased the number of NPCs and favored the fiber regeneration of neurons after stroke in the ischemia model by photothrombosis. There are two ways of neural stem cell proliferation: symmetrical and asymmetric division [31]. During the development of NPCs, they divide symmetrically to get a large number of nerve cells quickly and prepare for the growth and maturation of tissues and organs.

### 2.2. SCH Promotes In Vitro Proliferation of NPCs

**Figure 3:**
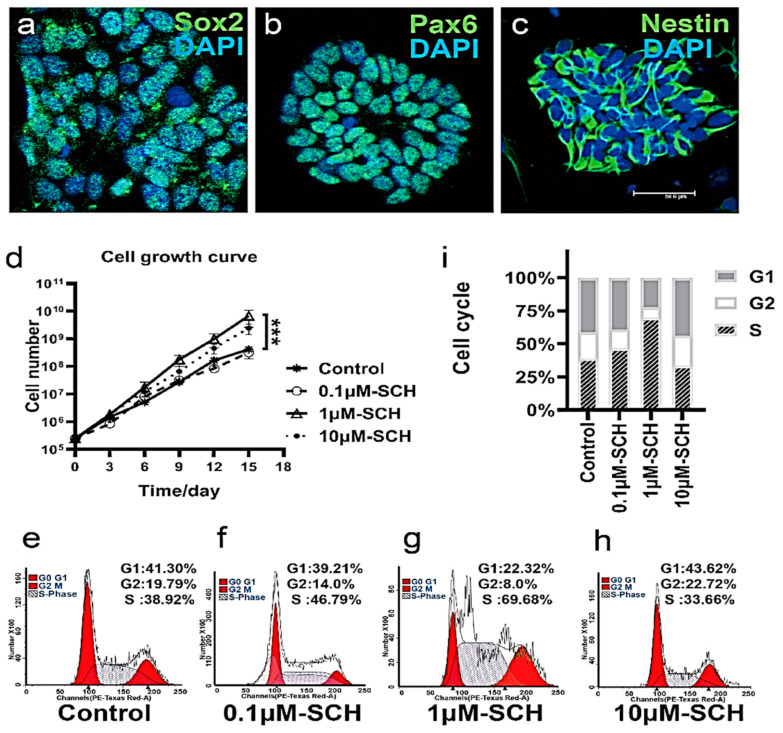
Effect of SCH on the proliferation of nerve progenitor cells in vitro. (**a**–**c**) nerve progenitor cell line (NSCs) BG02. (**a**) SOX2 (green) staining positive. (**b**) PAX6 (green) staining positive. (**c**) Nestin (green) staining positive. DAPI (blue) nuclear staining. Scale bars 75 µm. (**d**) Drawing growth Curve by 6 successive passages shows that SCH can increase the value more quickly. (**e**–**h**) Flow cytometry cell cycle analysis. (**i**) Cell cycle stacking map of different groups, SCH may promote the transformation of G1 to S phase. Count 20,000 cells.

NPCs were identified by staining specific markers of nestin, SOX2, PAX6 [32]. The results showed that all the cells we obtained were positive for nestin, Sox2, and Pax6. NPCs can be passed by more than 20 generations (Figure 3a–c). This suggests that neural epithelial stem cells have been obtained. We used the same batch of NPCs for different groups to control for differences. From the increment curve, we found that 1 μM SCH significantly promoted the proliferation of neural epithelial stem cells (Figure 3d), and more NPCs could be obtained after 72 h at the same number of inoculation (Figure 3d, 0.1 μM SCH: *p* = 0.9639; 1 μM SCH: *p* = 0.0006; 10 μM SCH: *p* = 0.005). The results showed that SCH could promote the proliferation of neurons under the maintenance culture of neural stem cells in vitro. In which, the asymmetric division of neural stem cells and NPCs differentiation into terminal cells could maintain the ability of nerve cells to be repaired against stroke and age-related diseases [3]. Furthermore, flow cytometry was used to detect cell cycle phases (Figure 3e–h). It was found that SCH could significantly increase the number of cells in the S phase. It is suggested that SCH may promote the transformation of the G1 to S phase (Figure 3i).

Cell polarization is associated with cell proliferation. Activation of the Rho type GTPases Cdc42 P is crucial for the establishment of cell polarity during G1 [33,34]. Because the humans and mice species are quite different, we have studied the increment effect of human NPCs cultured in vitro. The effect of SCH on the increase of NPCs was further demonstrated. SCH may promote cytoskeleton rearrangement by upregulating the expression of Cdc42, thus promoting cell polarization and transforming cells from G1 phase to S phase. Finally, more NPCs were found in ischemic cortical areas.

After brain ischemia, the synaptic remodeling of neurons was stimulated, and the activation of glial cells mediates the proliferation and differentiation of neural stem cells, but this was very weak and slow in change. Posterior penumbra or glial scar hinders the migration of nerve fibers and neurons to the ischemic and core areas. It has been reported that there are many signal pathways involved in cell remodeling and repair, cytoskeleton modification, and cell polarization. In general, these gene products promote changes in actin or microtubule cytoskeleton, which are associated with cell migration or axonal growth, especially in Rho and Ras pathways; axon-directed genes; cytoskeleton modification genes.

### 2.3. Effect of SCH on Differentiation

**Figure 4:**
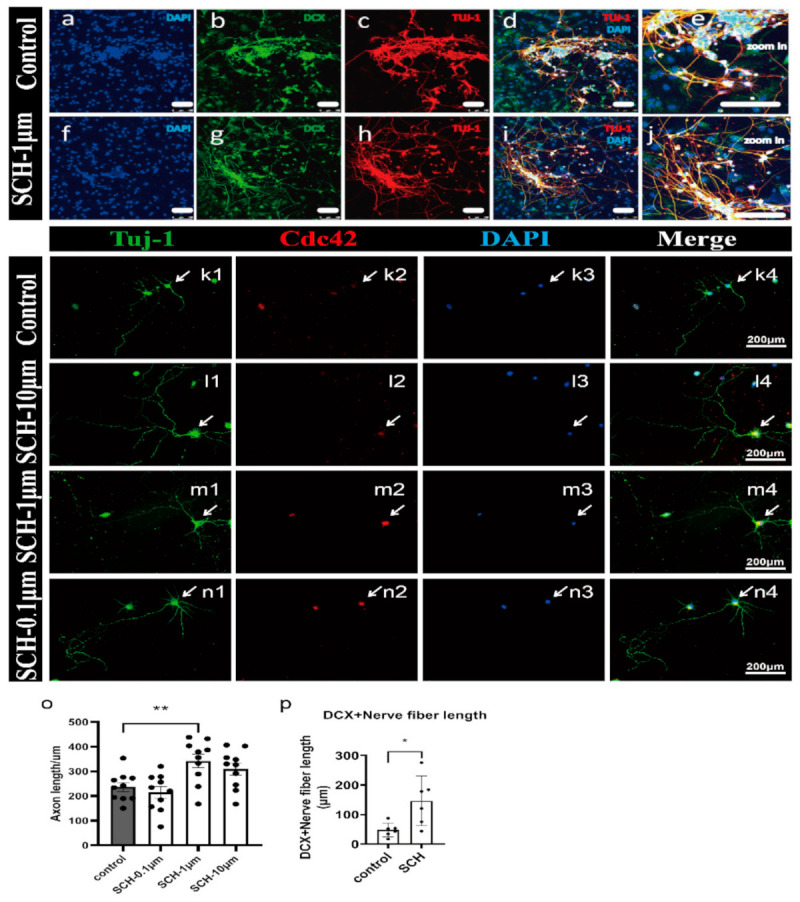
Effect of SCH on the Nerve fiber regeneration in NPCs *in vitro*. (**a**–**j**) neuronal differentiation of NPCs co-culture, scale bar 100 μm. (**a**,**f**) DAPI (bule); (**b**,**d**) Dcx (green). (**c**,**h**) Tuj-1 (red); (**d**,**i**) merged. (**p**) The nerve fibers in SCH group were longer than those in the control group. (**k**–**n**) Effects of SCH in Primary cortical neuron in vitro neuron with Tuj-1 and Cdc42 positive in SCH group. (**o**) Tuj-1 positive axon is longer in SCH group than the axon length in the control group. (**k2**–**n2**) The Cdc42 is positive in the SCH group neuron. The experiment was performed in three times. N = 10.* *p* < 0.05, ** *p* < 0.01, Data are expressed as the mean s. Scale bars: 200 μm.

The differentiation and maturation of neurons require the support and nutrition of astrocytes, so we used the method of co-culture astrocyte and NPCs to differentiate neurons. Tju-1 and DCX are two specific markers of neurons. The 1 μM SCH significantly promoted the maturation of neurons and had longer and denser axons than the control group (Figure 4a–j; *p* = 0.0203). Because we have figured out the best concentration of SCH, we just set up SCH treatment group. We did not just use NPC; we repeated the experiment on primary cortical neurons (Figure 4k–n). After 18 days of differentiation, the SCH group was formed by longer and denser axons than the control group. Compared with the control group, there were significant differences in SCH 1 μM and 10 μM (Figure 4o, *p* = 0.0094, *p* = 0.0941), and no significant difference in 0.1 μM SCH group (*p* = 0.8469). Both Tuj-1 and Cdc42 were expressed in these neurons at the same time, suggesting that neurofibrillary regeneration may be Cdc42 related (Figure 4k–n). Also, we found that SCH promoted the growth of axons and new nerve fibers into the penumbra by promoting the generation of active Cdc42. Furthermore, the expression of Cdc42 promoted by SCH in human NPCs cultured in vitro. This observation is in agreement with Takano et al. [35] who reported that the polarization of cell division (in which Cdc42 is involved in the formation of neurocortex) can underlie the directional flow of information in the central nervous system. Therefore, the establishment and maintenance of neuronal polarization are crucial for correct development and function.

### 2.4. Cdc42 Expression Level Analysis

**Figure 5:**
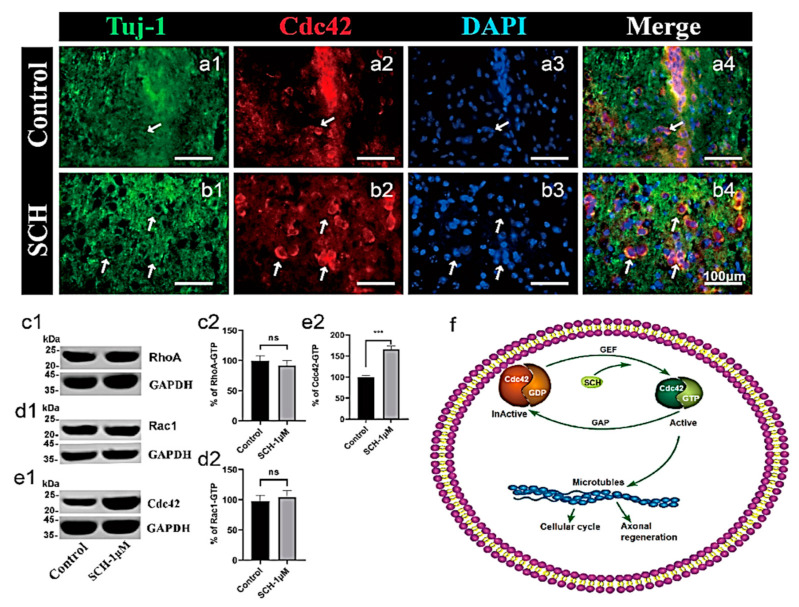
Effect of SCH on Cdc42 expression in vitro and in vivo. (**a**,**b**) Cdc42 expression levels in the ischemic area between SCH and control. There were some Cdc42 positive cells in the SCH group that the arrow points out, but it was negative in the control, scale bar: 100 μm. (**c**–**e**) Western blot assay for Cdc42, Rac1, and RhoA in NPCs. *** *p* < 0.001. Data are expressed as the mean Rho A. (**f**) The current model of Rho GTPase activation and signaling Regulation of Cdc42.

From stained brain slices, we found that Cdc42 expression was upregulated (Figure 5a,b). To determine the effects of other GTPase proteins, we use NPC cells to detect the effects of SCH on the expression of Cdc42, Rac1, and RhoA proteins in vitro and found that Cdc42 is the main acting protein (Figure 5c–e). There were significant differences in Cdc42 groups (Figure 5c2, *p* = 0.5029; d2, *p* = 0.6797; e2, *p* = 0.0001). Lastly, we suggest that SCH may promote the cell cycle and cytoskeletal rearrangement of NPCs by up-regulating the activity of Cdc42-GTP, and ultimately promote the number of neural stem cells and nerve fiber regeneration in the mice stroke model (Figure 5f). It was found that the migration of nerve fibers is related to the polarization of cells. In which, Rho family (GTPases, including Cdc42, Rac GTPases, and RhoA) have a central role in polarized growth processes [33].

Also, Cdc42 is involved in cytoskeleton formation, cell polarization, proliferation, and migration. Cdc42 activates the RAC signaling pathway and Rac activates Rho. The interaction between Cdc42 and actin is also involved in the process of cell polarization [36]. In which, in this study, we found that SCH may play an important role in promoting the growth of nerve fibers (Tuj-1, Map-2) through Cdc42. Rappaz, et al. [37] reported that Cdc42 is involved in the growth of neuronal axons and mediates the formation of the nerve fiber skeleton of f-actin.

## 3. Materials and Methods

### 3.1. Chemicals and Animals

Schisandrin A (Figure 1; CAS Number: 61281-38-6, molecular formula: C_24_H_32_O_6_, purity ≥ 98.0%, molecular mass: 416.507 Da) was purchased from Chengdu Institute of Biology (Chinese Academy of Sciences, Beijing, China). Paraformaldehyde (PFA), 4′,6-diamidino-2-phenylindole, dimethyl sulfoxide (DMSO), isoflurane, and Rose Bengal were purchased from Sigma Aldrich (New York, NY, USA).

Adult male C57/BL6 (6 mice per group), 10 weeks old of mice with weight 22–25 g (Shanghai SLAC Laboratory Animal, Shanghai, China) were used in this study to make sure that the mice are in the adult phase with a high probability of stroke formation. All the obtained animals were kept under standard conditions (temperature: 22 °C, light/dark cycle: 12/12 h) with unlimited access to water and food. Also, standard protocols and ethical approval were applied. This study was approved by the ethical committee for animal experiments of the Yunnan University of Chinese Medicine.

### 3.2. Induction of Ischemic Stroke and Preparation of Tissues for Photothrombosis Model Detection

Ischemia was induced by photothrombosis. Briefly, male mice (22–25 g) were anesthetized with isoflurane by inhalation technique. During the surgery, a feedback homoeothermic heating blanket was used to keep the mice’s body temperature.

The photothrombotic detection was measurement following the method of Galkov et al. [38] with some modifications. The scalp exposed area was located 0.2 mm in the right brain anterior-posterior (AP) and 1.5 mm of medial-lateral (ML) in relation to bregma. Within 2 min before the beginning of laser irradiation, Rose Bengal dye (1%; Sigma) was injected through a catheter placed into the jugular vein of ischemic animals at a dose of 1 mL/kg body weight, and an equivalent volume of saline was injected into control animals. The laser intensity was 1 mW and the irradiation time was 2 min. Then skins were sutured and the animals were raised for 3 weeks. Infarct areas were determined by Nissl’s through using a commercial kit with following the manufacturer’s protocol (Beyotime, Nanjing, China), at 48 h after the surgery. Also, staining by NeuN, Iba1, and GFAP were used to make sure the photothrombosis model was successfully implemented (Figure S1). In which, the core and penumbra area of six mice brains in each group were examined using 50 brain slices and the selection of the visualized photos were based on the largest infected area that was used for further analyses. The selected stained sections were examined and photographed under a microscope imaging system (Olympus Ltd., Tokyo, Japan). Cells with round shape, Nissl staining in cytoplasm, loose chromatin, and prominent nucleoli were considered as normal neurons; cells with shrunken shape, condensed or with no Nissl staining were considered as damaged neurons [39].

#### 3.2.1. Immunofluorescence Assay

The selected brains were cut into 20-μm coronal slices after 24 h of perfused with 4% paraformaldehyde following the method of Taccola, et al. [40] with some modifications. In brief, the slides were washed 3 times in PBS. Triton X-100 (1% BSA) was dripped onto the tissues for 2 h to prevent non-specific binding. Then primer antibodies were applied to the sections at a dilution of 1/100 and were incubated overnight at 37 °C. Slices were washed 3 times in PBS, followed by 2 h incubation at 37 °C with 1/250 of three types of secondary antibodies: goat anti-mouse IgG1(Y1), goat anti-Rabbit IgG (H + L), and goat anti-chicken IgG (H + L) (Invitrogen, Thermo Fisher Scientific, Shanghai, China) in 1% BSA. Nuclei were counterstained with 4′,6-diamidino-2-phenylindole (DAPI; 0.5 μg/mL; Sigma). Sections were mounted on a coverslip with glycerine. The pictures were obtained by Olympus BX61 fluorescence microscope equipped with CCD camera (Olympus Ltd.). The quantification analysis of the obtained Images’ cells numbers and intensities were by ImageJ software (version 1.53a, National Institutes of Health, Bethesda, MD, USA). Also, NeuronJ plugin of ImageJ software (National Institutes of Health) was used to track and analyze the axon’s length. Three wells were used and three fields at 20× were captured for each well (Figure S2).

#### 3.2.2. Cell Line Culture of NPCs and Primary Cortical Neuron Culture

NPCs were derived from embryonic stem cell line BG02, provided by Yunnan Key Laboratory of Primate Biomedical Research, Institute of Primate Translational Medicine [41]. NPCs culture medium contained Neurobasal medium (Gibco, Thermo Fisher Scientific), 1% N2 (Gibco), 2% B27 (Gibco), 1% NEAA (Sigma), 1% Glutmax (Sigma), 10 ng/mL bFGF (1:500; Millipore Ltd., Burlington, MA, USA), and 1000 U/mL LIF (Millipore), 3 μM CHIR99021 (Selleck, Houston, TX, USA), and 5 μM SB431542 (Cellagentec, San Diego, CA, USA). NPCs were cultured with 5 μg/mL laminin (Gibco) in poly-ornithine-coated 12-well plates and were passaged for every three days during the experiment [41]. Staining was carried out with PAX6 (1:500, Biolegend Covance, Dedham, MA, USA), SOX2 (1:500, Millipore), and Nestin (1:200, Millipore) [42], which are specific NSC markers. Primary cortical neuron culture was prepared from cerebral cortical tissue from postnatal day one (P1) wild-type C57BL/6 mice. The tissue was digested with 0.25% trypsin for 30 min followed by centrifugation at 1000 rpm for 5 min and filtering cells with 40 μm filter. Astrocytes culture medium contained DMEM (Gibco), 10% fetal bovine serum (FBS, Northvale, NJ, USA), and 1% NEAA (Sigma). Neuron culture medium contained Neurobasal (Gibco), 2% B27 (Gibco), 1% N2 (Gibco), and 1% NEAA (Sigma).

#### 3.2.3. Treatment with SCH

In vitro treatments were carried out using three different concentrations (0.1 μM, 1 μM, and 10 μM) of SCH in DMSO for treating the NPCs and primary neurons. Also, in vivo treatment with SCH consisted in intraperitoneal injection after 3 weeks from the first operation for ensuring the stability of the formed stroke. Duncan, et al. [43] reported that the initial intrinsic inflammatory response after stroke results in extensive cell death (apoptosis) up to one week after a stroke. In this acute phase, the brain is sensitive to further stress, and instability in its physical activity levels. Therefore, three weeks were used to make sure that SCH effect was stable after the regeneration process. SCH was dissolved in DMSO (30 mM) and administered at a dose of 12 mg/kg per day, for three weeks in a row [44]. The control group was injected with an equal volume of DMSO solution in PBS. The brain was collected after perfusion with 4% PFA.

#### 3.2.4. NPCs Cell Curve and Detection of Cell Cycle

NPCs were cultured in a 12-well plate that contained 2.5 × 10^5^ cells in each well. Cell count and passage were carried out every three days. Recording of six sampling points during continuous cell growth were used to produce the growth curve. In order to identify the cell cycle stage, cells were collected when the dishes reached 85% of their full capacity and digested with 0.05% trypsin (Sigma). Collected cells (2~5 × 10^6^) were fixed with 95% cold ethanol, filtered with 40 μm filter, and incubated with propidium iodide (Beyotime, Beijing, China) staining solution at 4 °C for 30 min. Detection of maximum excited wavelength was carried out at 488 nm by flow on-board. A total of 20,000 cells were counted to make flow cytometry.

#### 3.2.5. NPCs Differentiation Neurons in Co-Culture System

After differentiating NSCs in different media (SB431542, CHIR, LIF, bFGF, free medium) for three days, astrocytes were added to the plate. Immunofluorescence staining was performed 18 days later of cell differentiation. Three separate experiments were repeated three times. We used DCX (1:500; Millipore Ltd., Burlington, MA, USA) and Tuj-1 (1:500, Millipore Ltd., Burlington, MA, USA) to identify new neurons. We determined the differentiation of neurons by the length of their axons.

#### 3.2.6. Western Blot Assay

Total protein was extracted from 6 well-cultured plate of NPCs. Cells were then lysed with 4× laemmli sample buffer (BIO-RAD, Hercules, CA, USA), 1 mM DTT. Proteins were separated by 12% sodium dodecyl sulfate-polyacrylamide gel electrophoresis (SDS-PAGE) and then transferred onto polyvinylidene fluoride membranes (Neobioscience Biotech, Beijing, China). The membranes were blocked with 5% nonfat milk in Tris-buffered saline containing 0.1% Tween-20 (TBST) for 1 h and incubated overnight with primary antibody at 4 °C. That solution included RhoA (1:500 mouse, Santa Cruz, Shanghai, China), Rac1 (1:1000 mouse, BD Transduction Laboratories, Shanghai, China), Cdc42 (1:250, mouse, Santa Cruz), and GAPDH (1:1000, mouse, HuaBio, Shanghai, China). Then, it was washed three times in tris buffer saline with TBST. After that, it was incubated with 1 μg/mL of horseradish peroxidase-linked goat-anti-rabbit antibody (IgG; Huabio Inc., Beijing, China) and 1 μg/mL of goat-anti-mouse antibody (IgG; Huabio Inc., Beijing, China) for 1 h at 37 °C. Blots were visualized using the ECL chemiluminescent system (Amersham Imager 600, GE Healthcare, Tokyo, Japan) and quantified using the ImageJ software (version 1.53a, National Institutes of Health, Bethesda, MD, USA).

### 3.3. Statistical Analysis

Data are presented as the mean ± SEM, and statistics using GraphPad Prism (San Diego, CA, USA). Analysis of variance (ANOVA) was applied to detect the significant difference and *p* ≤ 0.05 was considered statistically significant and *p* ≤ 0.01 was considered as highly significant. Duncan and Pearson’s tests were calculated to measure the significance among the tested groups and properties. Cutting and processing of pictures using ImageJ software.

## 4. Conclusion

There are very few chemical drugs that can be used therapeutically for a long time after a stroke. These chemicals have several negative side effects and human toxicity. The present study indicated that SCH, a natural safe phytochemical, has the potential key properties for promoting NPC proliferation in human neural stem cells and mice models. In comparison with the control group, the SCH group had more MAP-2 positive nerve axon regeneration in the penumbra and core. It promoted the skeletal rearrangement through the Cdc42 and GTPase proteins that enhance neuronal axon development. Also, 1 μM SCH significantly promoted the proliferation of neural epithelial stem cells with increased maturation of neurons compared to the control group. Therefore, the results of the study suggest the use of SCH for promoting the regeneration of NPCs in the later stage of stroke disease. Also, the potential application of SCH in medicinal foods may enhance their lifestyle protective impact the neural diseases like Alzheimer’s.

## Data Availability

Not applicable.

## Supplementary Materials

The following are available online; Figure S1. (**a1**) NeuN loss of brain ischemic area (reddish grey) at 48 h at Scale 1 mm. (**a2**) A higher magnification image of stroke area. (**b1**) Enhanced GFAP expression in the transition zone between ischemic core (Iba1+) and penumbra (GFAP+) after photothrombosis. (**b2**) A higher magnification image of between ischemic core (Iba1+) and penumbra (GFAP+) after photothrombosis. (**c1**) Enhanced DCX expression in the ischemic area after photothrombosis. (**c2**) A higher magnification image of the enhanced DCX expression in the ischemic area. (**d1**) Enhanced SOX2 expression in the ischemic area after photothrombosis, NeuN (red), DAPI (bule). (**d2**) A higher magnification image of SOX2 expression in the ischemic area. Figure sup2. NeuronJ-ImageJ software was used to track and analysis the axons length. (**a1**) Neuron staining by NeuN (red), Tuj-1 (green). (**a2**) Convert the picture to 8-bits. (**a3**) tracking the axons with NeuronJ-ImageJ software (purple). (**a4**) Axon tracking is shown separately. Scale bars 50 µm.

## Sample Availability

Sample of SCH is available from the authors.

## References

[1] Pandian J.D., Kalkonde Y., Sebastian I.A., Felix C., Urimubenshi G., Bosch J. (2020). Stroke systems of care in low-income and middle-income countries: Challenges and opportunities. Lancet.

[2] Kahroba H., Ramezani B., Maadi H., Sadeghi M.R., Jaberie H., Ramezani F. (2021). The role of nrf2 in neural stem/progenitors cells: From maintaining stemness and self-renewal to promoting differentiation capability and facilitating therapeutic application in neurodegenerative disease. Ageing Res. Rev..

[3] Jodeiri Farshbaf M., Alvina K. (2021). Multiple roles in neuroprotection for the exercise derived myokine irisin. Front. Aging Neurosci..

[4] Lee H.J., Ahn S.M., Pak M.E., Jung D.H., Lee S.Y., Shin H.K., Choi B.T. (2018). Positive effects of alpha-asarone on transplanted neural progenitor cells in a murine model of ischemic stroke. Phytomedicine Int. J. Phytother. Phytopharm..

[5] Liu L., Zhao X., Silva M., Li S., Xing X., Zheng W. (2020). Artemisinin protects motoneurons against axotomy-induced apoptosis through activation of the pka-akt signaling pathway and promotes neural stem/progenitor cells differentiation into neun(+) neurons. Pharmacol. Res..

[6] Zamboni M., Llorens-Bobadilla E., Magnusson J.P., Frisén J. (2020). A widespread neurogenic potential of neocortical astrocytes is induced by injury. Cell Stem Cell.

[7] Yu X., Qi Y., Zhao T., Fang J., Liu X., Xu T., Yang Q., Dai X. (2019). Ngf increases fgf2 expression and promotes endothelial cell migration and tube formation through pi3k/akt and erk/mapk pathways in human chondrocytes. Osteoarthr. Cartil..

[8] Hussein L., Gouda M., Buttar H.S. (2021). Pomegranate, its components and modern deliverable formulations as potential botanicals in the prevention and treatment of various cancers. Curr. Drug Deliv..

[9] Gouda M., Huang Z., Liu Y., He Y., Li X. (2021). Physicochemical impact of bioactive terpenes on the microalgae biomass structural characteristics. Bioresour. Technol..

[10] Ahmed F.E., Gouda M.M., Hussein L.A., Ahmed N.C., Vos P.W., Mohammad M.A. (2017). Role of melt curve analysis in interpretation of nutrigenomics’ microrna expression data. Cancer Genom. Proteom..

[11] Gouda M., El-Din Bekhit A., Tang Y., Huang Y., Huang L., He Y., Li X. (2021). Recent innovations of ultrasound green technology in herbal phytochemistry: A review. Ultrason. Sonochemistry.

[12] Lv J.M., Gouda M., Zhu Y.Y., Ye X.Q., Chen J.C. (2021). Ultrasound-assisted extraction optimization of proanthocyanidins from kiwi (actinidia chinensis) leaves and evaluation of its antioxidant activity. Antioxidants.

[13] Cai N.N., Wang Z.Z., Zhu X.C., Jiang Y., Zhu W.Q., Yang R., Zhang X.M. (2020). Schisandrin a and b enhance the dentate gyrus neurogenesis in mouse hippocampus. J. Chem. Neuroanat..

[14] Liu X., Cong L., Wang C., Li H., Zhang C., Guan X., Liu P., Xie Y., Chen J., Sun J. (2019). Pharmacokinetics and distribution of schisandrol a and its major metabolites in rats. Xenobiotica.

[15] Szopa A., Ekiert R., Ekiert H. (2017). Current knowledge of schisandra chinensis (turcz.) baill. (chinese magnolia vine) as a medicinal plant species: A review on the bioactive components, pharmacological properties, analytical and biotechnological studies. Phytochem. Rev. Proc. Phytochem. Soc. Eur..

[16] Jin X., Guo J.L., Wang L., Zhong X., Yao W.F., Gao H., Liu M.Y. (2021). Natural products as pharmacological modulators of mitochondrial dysfunctions for the treatments of alzheimer’s disease: A comprehensive review. Eur. J. Med. Chem..

[17] Zhi Y., Jin Y., Pan L., Zhang A., Liu F. (2019). Schisandrin a ameliorates mptp-induced parkinson’s disease in a mouse model via regulation of brain autophagy. Arch. Pharmacal Res..

[18] Bao J., Zhang Y., Zhang L., Gong X., Shi W., Liu L., Wang X. (2021). Therapeutic effect of schisandrin a on avian colibacillosis through gut-liver axis. Poult. Sci..

[19] Wan M.L.Y., Turner P.C., Co V.A., Wang M.F., Amiri K.M.A., El-Nezami H. (2019). Schisandrin a protects intestinal epithelial cells from deoxynivalenol-induced cytotoxicity, oxidative damage and inflammation. Sci. Rep..

[20] Qi Y., Cheng X., Jing H., Yan T., Xiao F., Wu B., Bi K., Jia Y. (2019). Combination of schisandrin and nootkatone exerts neuroprotective effect in alzheimer’s disease mice model. Metab. Brain Dis..

[21] Li Z., Zhao L., Xia Y., Chen J., Hua M., Sun Y. (2021). Schisandrin b attenuates hepatic stellate cell activation and promotes apoptosis to protect against liver fibrosis. Molecules.

[22] Buscemi L., Price M., Bezzi P., Hirt L. (2019). Spatio-temporal overview of neuroinflammation in an experimental mouse stroke model. Sci. Rep..

[23] Lindvall O., Kokaia Z. (2015). Neurogenesis following stroke affecting the adult brain. Cold Spring Harb. Perspect. Biol..

[24] Cheng A.H., Bouchard-Cannon P., Hegazi S., Lowden C., Fung S.W., Chiang C.K., Ness R.W., Cheng H.M. (2019). Sox2-dependent transcription in clock neurons promotes the robustness of the central circadian pacemaker. Cell Rep..

[25] Biswas S., Chung S.H., Jiang P., Dehghan S., Deng W. (2019). Development of glial restricted human neural stem cells for oligodendrocyte differentiation in vitro and in vivo. Sci. Rep..

[26] Ma G., Abbasi F., Koch W.T., Mostowski H., Varadkar P., McCright B. (2018). Evaluation of the differentiation status of neural stem cells based on cell morphology and the expression of notch and sox2. Cytotherapy.

[27] Jimenez-Gonzalez A., Garcia-Concejo A., Leon-Lobera F., Rodriguez R.E. (2018). Morphine delays neural stem cells differentiation by facilitating nestin overexpression. Biochim. et Biophys. Acta. Gen. Subj..

[28] Spijkers X.M., Pasteuning-Vuhman S., Dorleijn J.C., Vulto P., Wevers N.R., Pasterkamp R.J. (2021). A directional 3d neurite outgrowth model for studying motor axon biology and disease. Sci. Rep..

[29] Lin Y., Zhang J.C., Yao C.Y., Wu Y., Abdelgawad A.F., Yao S.L., Yuan S.Y. (2016). Critical role of astrocytic interleukin-17 a in post-stroke survival and neuronal differentiation of neural precursor cells in adult mice. Cell Death Dis..

[30] Zhang B., Li F., Zhao W., Li J., Li Q., Wang W. (2015). Protective effects of allicin against ischemic stroke in a rat model of middle cerebral artery occlusion. Mol. Med. Rep..

[31] Dranovsky A., Picchini A.M., Moadel T., Sisti A.C., Yamada A., Kimura S., Leonardo E.D., Hen R. (2011). Experience dictates stem cell fate in the adult hippocampus. Neuron.

[32] Zhao C., Deng W., Gage F.H. (2008). Mechanisms and functional implications of adult neurogenesis. Cell.

[33] Govek E.E., Wu Z., Acehan D., Molina H., Rivera K., Zhu X., Fang Y., Tessier-Lavigne M., Hatten M.E. (2018). Cdc42 regulates neuronal polarity during cerebellar axon formation and glial-guided migration. iScience.

[34] Quadri R., Galli M., Galati E., Rotondo G., Gallo G.R., Panigada D., Plevani P., Muzi-Falconi M. (2020). Haspin regulates ras localization to promote cdc24-driven mitotic depolarization. Cell Discov..

[35] Takano T., Xu C., Funahashi Y., Namba T., Kaibuchi K. (2015). Neuronal polarization. Development.

[36] Xu X.-P., He H.-L., Hu S.-L., Han J.-B., Huang L.-L., Xu J.-Y., Xie J.-F., Liu A.-R., Yang Y., Qiu H.-B. (2017). Ang ii-at2r increases mesenchymal stem cell migration by signaling through the fak and rhoa/cdc42 pathways in vitro. Stem Cell Res. Ther..

[37] Rappaz B., Lai Wing Sun K., Correia J.P., Wiseman P.W., Kennedy T.E. (2016). Flim fret visualization of cdc42 activation by netrin-1 in embryonic spinal commissural neuron growth cones. PLoS ONE.

[38] Galkov M., Gulyaev M., Kiseleva E., Andreev-Andrievskiy A., Gorbacheva L. (2020). Methods for detection of brain injury after photothrombosis-induced ischemia in mice: Characteristics and new aspects of their application. J. Neurosci. Methods.

[39] Jin W., Xu W., Chen J., Zhang X., Shi L., Ren C. (2016). Remote limb preconditioning protects against ischemia-induced neuronal death through ameliorating neuronal oxidative DNA damage and parthanatos. J. Neurol. Sci..

[40] Taccola C., Barneoud P., Cartot-Cotton S., Valente D., Schussler N., Saubamea B., Chasseigneaux S., Cochois V., Mignon V., Curis E. (2021). Modifications of physical and functional integrity of the blood-brain barrier in an inducible mouse model of neurodegeneration. Neuropharmacology.

[41] Zhu X., Li B., Ai Z., Xiang Z., Zhang K., Qiu X., Chen Y., Li Y., Rizak J.D., Niu Y. (2016). A robust single primate neuroepithelial cell clonal expansion system for neural tube development and disease studies. Stem Cell Rep..

[42] Kumamaru H., Kadoya K., Adler A.F., Takashima Y., Graham L., Coppola G., Tuszynski M.H. (2018). Generation and post-injury integration of human spinal cord neural stem cells. Nat. Methods.

[43] Duncan P.W., Min Lai S., Keighley J. (2000). Defining post-stroke recovery: Implications for design and interpretation of drug trials. Neuropharmacology.

[44] Sun Y.-X., Cong Y.-L., Liu Y., Jin B., Si L., Wang A.-B., Cai H., Che G.-Y., Tang B., Wang C.-F. (2014). Schisandrin a and b affect subventricular zone neurogenesis in mouse. Eur. J. Pharmacol..

