# Supplementary material for: The Antioxidant Phytochemical Schisandrin A Promotes Neural Cell Proliferation and Differentiation after Ischemic Brain Injury
Source: Molecules. 2021 Dec 9;26(24):7466. doi: 10.3390/molecules26247466 (PMC8706049; doi:10.3390/molecules26247466)
Supplement: Supplementary file 1 [file molecules-26-07466-s001.zip › molecules-1516111-supplementary.pdf]

Supplementary Material

The Antioxidant Phytochemical Schisandrin A Promotes Neural Cell Proliferation and Differentiation after Ischemic Brain Injury

Wentian Zong [1], Mostafa Gouda [2,3,*], Enli Cai [4], Wang Ruofeng [5], Xu Weijie [1], Yuming Wu [4,*], Paulo E. S. Munekata [6,*] and José M. Lorenzo [6,7]

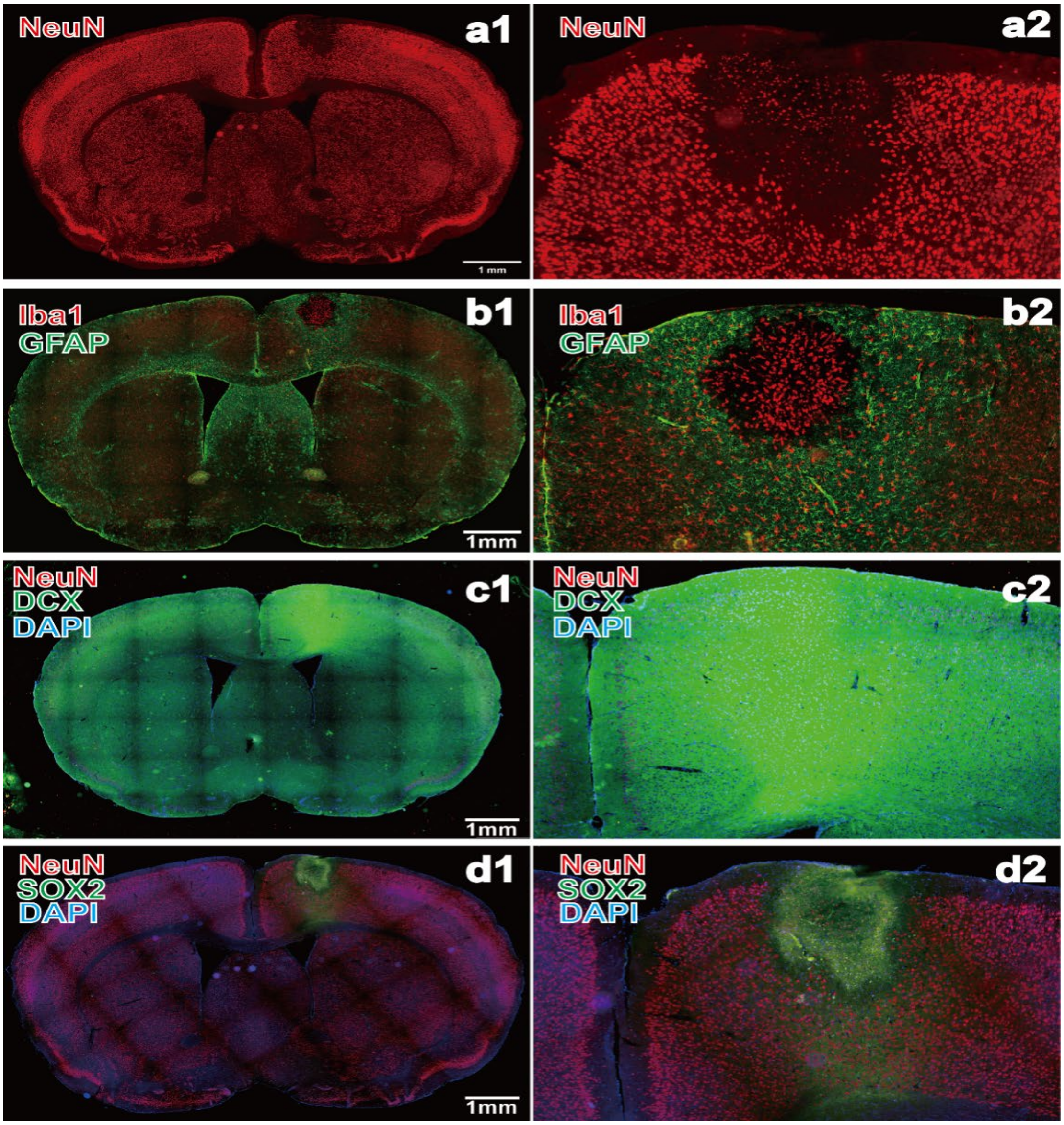

Figure S1. (a1) NeuN loss of brain ischemic area (reddish grey) at 48h at Scale 1mm). (a2) A higher magnification image of stroke area. (b1) Enhanced GFAP expression in the transition zone between ischemic core（Iba1+）and penumbra（GFAP+） after photothrombosis. (b2) A higher magnification image of between ischemic core（Iba1+）and penumbra（GFAP+） after photothrombosis. (c1) Enhanced

DCX expression in the ischemic area after photothrombosis. (c2) A higher magnification image of the enhanced DCX expression in the ischemic area. (d1) Enhanced SOX2 expression in the ischemic area after photothrombosis, NeuN (red), DAPI (bule). (d2) A higher magnification image of SOX2 expression in the ischemicarea.

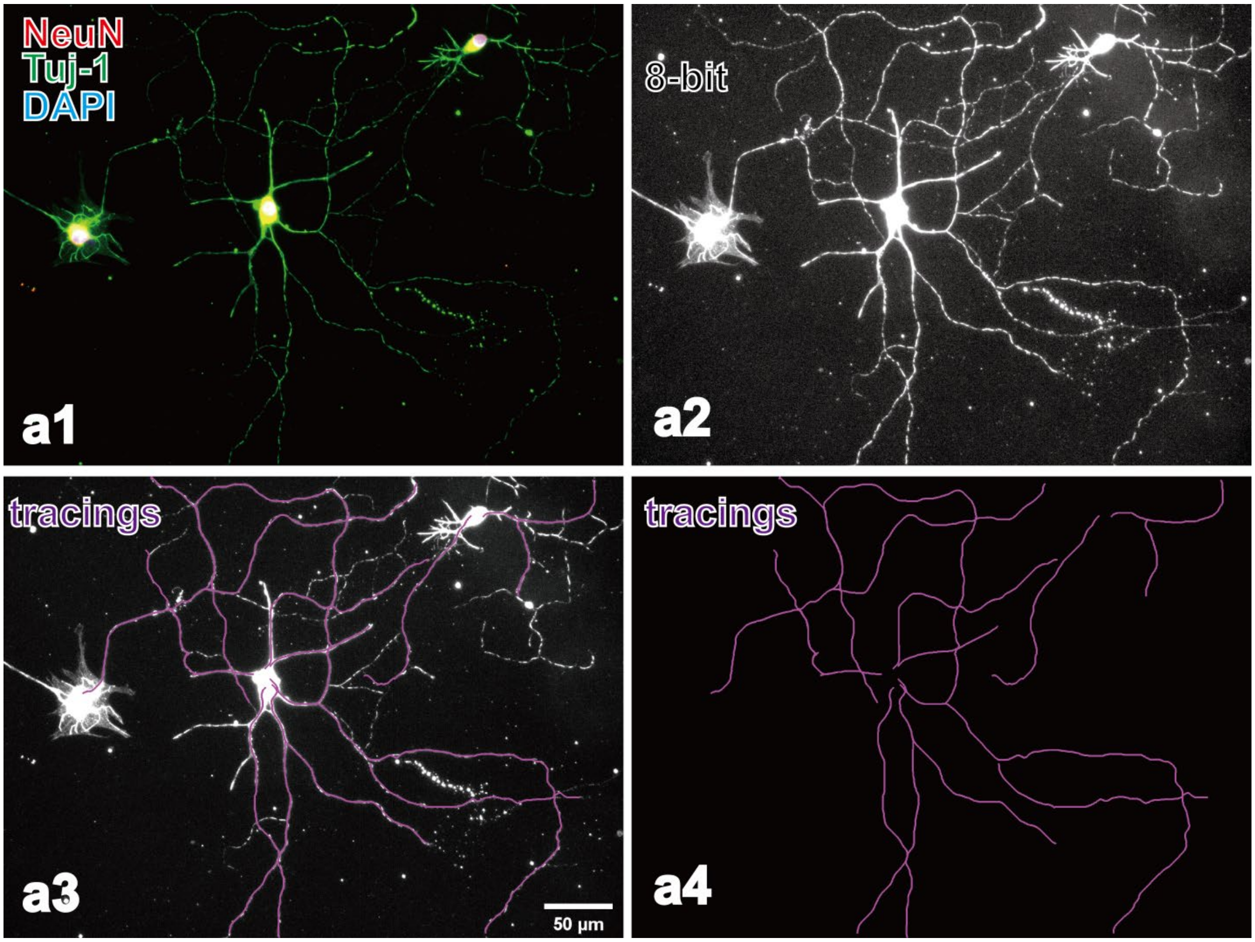

Figure S2. NeuronJ - ImageJ software was used to track and analysis the axons length. (a1) Neuron staining by NeuN(red), Tuj-1(green) . (a2) Convert the picture to 8-bits. (a3) tracking the axons with NeuronJ - ImageJ software (purple) .(b4)Axon tracking is shown separately. Scale bars 50 μm.
